# FIB‐3 index as a novel age‐independent predictor of liver fibrosis and prognosis in hepatocellular carcinoma patients undergoing hepatectomy

**DOI:** 10.1002/ags3.70010

**Published:** 2025-04-13

**Authors:** Yuki Imaoka, Masahiro Ohira, Tsuyoshi Kobayashi, Naruhiko Honmyo, Michinori Hamaoka, Takashi Onoe, Daisuke Takei, Koichi Oishi, Tomoyuki Abe, Toshihiro Nakayama, Miho Akabane, Kazunari Sasaki, Hideki Ohdan, Hiroshima Surgical study group of Clinical Oncology (HiSCO)

**Affiliations:** ^1^ Department of Gastroenterological and Transplant Surgery, Graduate School of Biomedical and Health Sciences Hiroshima University Hiroshima University Hiroshima Japan; ^2^ Division of Abdominal Transplant Stanford University Stanford California USA; ^3^ Division of Regeneration and Medicine, Medical Center for Translational and Clinical Research Hiroshima University Hospital Hiroshima Japan; ^4^ Department of Surgery Hiroshima City North Medical Center Asa Citizens Hospital Hiroshima Japan; ^5^ Department of Gastroenterological Breast and Transplant Surgery Hiroshima Prefectural Hospital Hiroshima Japan; ^6^ Department of Surgery National Hospital Organization Kure Medical Center and Chugoku Cancer Center Kure City Japan; ^7^ Department of Surgery Onomichi General Hospital Onomichi City Japan; ^8^ Department of Surgery Chugoku Rosai Hospital Kure City Japan; ^9^ Department of Surgery National Hospital Organization Higashihiroshima Medical Center Higashihiroshima City Japan

**Keywords:** FIB3‐index, hepatectomy, hepatocellular carcinoma, liver fibrosis

## Abstract

**Background:**

Liver fibrosis is a key factor in the progression of chronic liver diseases, including viral hepatitis and metabolic dysfunction‐associated steatotic liver disease. If untreated, fibrosis can progress to cirrhosis, increasing the risk of liver cancer or failure. This study evaluates the Fibrosis (FIB)‐3 index, a novel marker free from age‐related biases, for predicting liver fibrosis and 5‐year outcomes in hepatocellular carcinoma (HCC) patients undergoing hepatectomy.

**Methods:**

Data from 1013 patients who underwent liver resection were analyzed in this multi‐institutional study. The predictive performance of the FIB‐3 index was compared with the original FIB‐4 index, which incorporates age into its calculation.

**Results:**

The FIB‐3 index demonstrated superior accuracy for advanced fibrosis (≥F3) in elderly patients. A higher FIB‐3 index was an independent risk factor for recurrence‐free survival in elderly patients, underscoring its utility in this population. Notably, the application of appropriate cutoff values allowed the FIB‐3 index to facilitate effective risk stratification for 5‐year overall survival and recurrence‐free survival.

**Conclusions:**

The FIB‐3 index served as an effective alternative to the FIB‐4 index in assessing liver fibrosis among aged patients, and it effectively stratified the likelihood of the 5‐year outcomes when utilized in conjunction with a specific cut‐off after initial hepatectomy for HCC.

## Graphical abstract

Fibrosis‐3 index (FIB‐3) can stratify the risk of the survival and the recurrence‐free survival. The FIB‐3 index is a reliable, age‐independent tool for assessing liver fibrosis and prognosis in HCC patients post‐hepatectomy. Its use may improve clinical outcomes and support personalized treatment strategies.

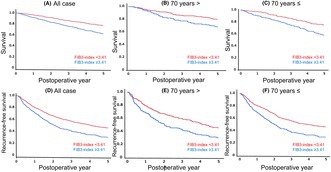

## 1 INTRODUCTION

Liver fibrosis is a significant contributor to the progression of a plethora of chronic liver diseases, including viral hepatitis infection and metabolic dysfunction‐associated steatotic liver disease (MASLD).^1^ If left untreated, liver fibrosis can advance to liver cirrhosis (LC), which may increase the risk of multicentric carcinogenesis or failure.^2^ In particular, after liver resection for HCC patients with LC exhibit a heightened annual risk of de novo recurrence, ranging from 6% to 15%, compared to patients with a normal liver.^3^ Notably, several studies have established a correlation between the noninvasive fibrosis marker, Fibrosis‐4 (FIB‐4) index, and poor overall survival (OS) and recurrence‐free survival (RFS) in patients who have undergone hepatectomy for HCC.^4^, ^5^ Recently, a weakness of the FIB‐4 index has come to light that it tends to overestimate fibrosis levels in the elderly.

Consequently, a new index, the FIB‐3 index,^6^, ^7^ was created to replace the FIB‐4 index, without an age component. The diagnostic ability of the FIB‐3 index for liver fibrosis has been verified, but its ability to predict prognosis after HCC resection has not been sufficiently verified. The proportion of elderly patients undergoing liver resection is on the rise.^8^ In addition, the approval of an Interferon‐free Direct‐Acting Antiviral (DAA) has coincided with changes in the epidemiology of liver resection for HCC, as evidenced by the increasing proportion of Non‐B, Non‐C Hepatitis (NBNC) cases.^9^ Considering recent trends, it is expected that the importance of non‐invasive liver fibrosis markers that can predict potential de‐novo recurrence risk and a poor prognosis risk will increase in liver resections for HCC caused by various liver diseases and in liver resections in the elderly.

The study aimed to evaluate the predictive ability of the FIB‐3 index for liver fibrosis and 5‐year prognosis in patients undergoing liver resections for HCC.

## 2 MATERIALS AND METHODS

### 2.1 Patients

A total of 1361 initial hepatectomies for primary HCC were performed by the Hiroshima Surgical Study Group of Clinical Oncology (HiSCO) between 2010 and 2018. The analysis included all 1013 cases that underwent liver resection, excluding those with insufficient data and cases in which R0 surgery was not achieved. The adaptation of liver resection was based on a published flow chart that considered the presence of ascites, serum total bilirubin levels, and indocyanine green retention rate at 15 min (ICG‐R15).^10^ Clinicodemographic data such as age, sex, body mass index (BMI), Child–Pugh classification, hepatitis virus type B (HBV) surface antigen, HCV antibody, pathological findings, preoperative blood tests, and tumor markers (e.g., alpha‐fetoprotein and des‐gamma‐carboxyprothrombin) were obtained from electronic records. Additionally, the rates of HCC recurrence and long‐term survival after surgery were obtained from clinical records.

Following hepatectomy, patients were monitored using ultrasound, contrast‐CT, or MRI, combined with an evaluation of serum levels of alpha‐fetoprotein and des‐gamma‐carboxyprothrombin at 3‐month intervals for up to 3 years and 6‐month intervals for up to 5 years thereafter. Diagnosis was histologically confirmed when necessary. Postoperative management and postoperative recurrence treatment were performed in accordance with the HCC guidelines at all facilities.^11^

### 2.2 Definition of fibrosis stage and blood‐based markers for liver fibrosis

The stage of liver fibrosis (F0 to F4) was diagnosed based on pathological findings using the scoring system proposed. The definition of each fibrosis is as follows: F0; no fibrosis, F1; fibrous portal expansion, F2; bridging fibrosis (portal‐portal or portal‐central linkage), F3; bridging fibrosis with lobular distortion, F4; cirrhosis.^12^

The FIB‐4 index, originally formulated for patients co‐infected with human immunodeficiency virus (HIV) and HCV, was derived using Sterling's formula^13^ as follows:

$$\mathrm{FIB}-4\;\text{index}=\mathrm{Age}\;\left(\text{years}\right)\times \mathrm{AST}\;\left(U/L\right)/\left(\mathrm{PLT}\;\left[10^{9}/L\right]\times \surd \mathrm{ALT}\;\left[U/L\right]\right)$$

The FIB‐3 indicator is the FIB‐4 indicator minus the age component. The calculation formula of the FIB‐3 index^6^, ^7^ is as follows:

$$\mathrm{FIB}-3\;\text{index}=5\times \ln^{*}\;\left(\mathrm{AST}\left[U/L\right]\right)-2\times \ln\;\left(\mathrm{ALT}\left[U/L\right]\right)-0.18\times \left(\mathrm{PLT}\;\left[10^{4}/\mathrm{\mu L}\right]\right)$$

*ln(*x*) represents the natural logarithm of *x*. The cut‐off values of the FIB‐3 index and FIB‐4 index reported in the previous study^7^ were identified as 3.41 and 2.01, respectively.

### 2.3 Statistical analysis

Comparative analyses between independent groups were performed using the nonparametric Mann–Whitney *U* test. The *p*‐value threshold was set at *p* < 0.05, indicating statistical significance. Data were reported as median values with minimum and maximum ranges. Survival curves were generated using Kaplan–Meier estimations and contrasted using log‐rank tests. Comparative evaluations of different prognostic frameworks considered metrics such as the Area Under the Receiver Operating Characteristic curve (AUROC). AUROC comparisons between models for predicting liver fibrosis and postoperative outcomes were performed using the DeLong test. The accuracy of liver fibrosis prediction between the two models was compared using McNemar's test. Multivariate analyses were performed with all variables to detect independently related risk factors to the 5‐year OS and RFS using the Cox proportional hazard model. To adjust for differences in baseline characteristics, one‐to‐one propensity score models were constructed on the basis of each patient's estimated propensity score based on the identified independence risk factors for the 5‐year RFS using the multivariate cox regression analysis. All statistical procedures were performed using the JMP® statistical software (Version 18; SAS Institute Inc., Cary, NC, USA).

## 3 RESULTS

### 3.1 Correlation between FIB3‐index and clinical characteristics

**FIGURE 1:**
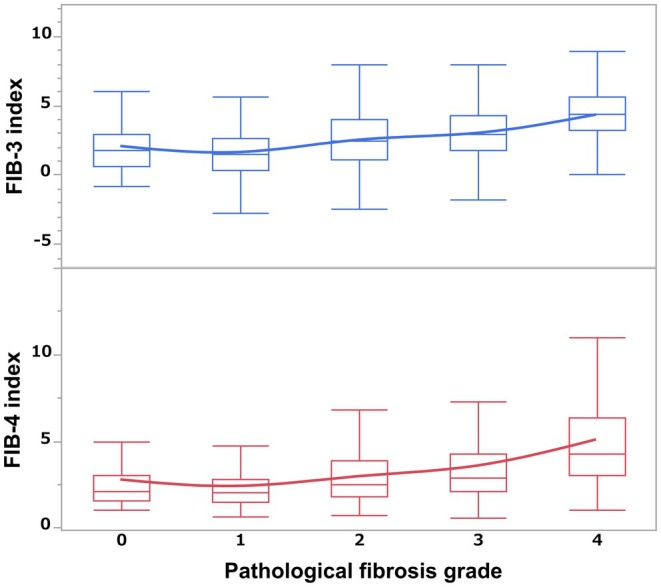
The noninvasive liver fibrosis markers and pathological liver fibrosis grade. The distribution of the FIB‐3 and FIB‐4 indices and the pathological liver fibrosis grade are shown in the figure.

This study included 1013 patients, including 807 men (79.6%) and 206 women (21.4%). The median age of the patient cohort was 72 years [65–78], and the median total tumor size was 26 mm [18–40]. As shown in Figure 1, the median FIB‐3 index for each fibrosis grade was 1.78 [0.62–0.98] for grade 0, 1.53 [0.36–2.64] for grade 1, 2.49 [1.13–3.98] for grade 2, 2.98 [1.76–4.33] for grade 3, and 4.43 [3.21–5.62] for grade 4. The median FIB‐4 index for each fibrosis grade was 2.09 [1.56–3.02] for grade 0, 2.06 [1.52–2.81] for grade 1, 2.49 [1.76–3.85] for grade 2, 2.86 [2.14–4.25] for grade 3, and 4.30 [3.04–6.36] for grade 4.

Patient stratification was conducted according to the FIB‐3 index into the low group (<3.41, *N* = 603) and high group (≥3.41, *N* = 410) (Table 1). Significant differences were noted in the FIB‐3 index, FIB‐4 index, T‐bil, AST, ALT, Platelet count, Albumin, and ICG‐R15, with worse values in the high FIB‐3 index group (*p* < 0.01). AFP levels were significantly higher in the high FIB‐3 index group (*p* < 0.01), whereas DCP levels were not significantly different (*p* = 0.36). The median tumor size was smaller in the high FIB‐3 index group (25 mm) compared to the low FIB‐3 index group (28 mm) (*p* < 0.01). The high FIB‐3 index group had a significantly higher proportion of patients with multiple tumors (29.0% vs. 22.6%, *p* = 0.02). On the other hand, there were no significant differences among intrahepatic metastasis (im), vascular invasion, and the presence of poor differentiation. The low FIB‐3 index group had a significantly higher rate of intrahepatic recurrence and early recurrence within 2 years after resection.

**TABLE 1:** Background data due to the FIB‐3 index.

Subject	Low FIB‐3 index <3.41 *N* = 603	High FIB‐3 index ≥3.41 *N* = 410	*p*‐Value
Gender, male	525 (87.1)	282 (68.8)	<0.01
Age, years	71 [65–77]	72 [66–78]	0.12
HBV, HCV, B + C, NBNC, Alcohol	132/224/5/194/48	46/239/5/88/32	<0.01
Body mass index	23.3 [21.1–25.4]	23.0 [20.8–25.3]	0.35
*Laboratory data*			
Child–Pugh A, yes	591 (98.0)	361 (88.1)	<0.01
Liver Damage Grade A, yes	505 (83.8)	237 (57.8)	<0.01
FIB‐3 index	1.78 [0.75–2.62]	4.83 [4.13–5.84]	<0.01
FIB‐4 index	2.12 [1.59–2.72]	4.88 [3.89–6.51]	<0.01
T‐bil, mg/dL	0.7 [0.6–0.9]	0.8 [0.6–1.0]	<0.01
AST, IU/mL	26 [21–33]	47 [36–64]	<0.01
ALT, IU/mL	23 [16–36]	36 [24–55]	<0.01
Platelet number, 10^9^/L	182 [149–232]	113 [86–147]	<0.01
Albumin, g/L	41 [38–44]	39 [35–42]	<0.01
ICG‐R15, %	10.8 [7.4–16.1]	17.4 [11.9–24.5]	<0.01
DCP, mAU/mL	62 [24–476]	56 [22–347]	0.36
AFP, ng/mL	5.9 [3.0–38]	12.8 [5.3–78]	<0.01
*Operative factor*			
Anatomical resection	451 (74.8)	240 (58.5)	<0.01
Operation time, min	283 [216–358]	284 [208–353]	0.20
Bleeding volume, mL	270 [120–550]	300 [130–610]	0.07
*Pathological factor*			
Tumor size, mm	28 [19–43]	25 [17–37]	<0.01
Multiple tumors, yes	136 (22.6)	119 (29.0)	0.02
Intrahepatic metastasis, yes	61 (10.1)	47 (11.5)	0.50
Vascular invasion, yes	189 (31.3)	140 (34.1)	0.35
Poor differentiated	67 (11.1)	40 (9.8)	0.56
*Recurrence pattern*			
Intrahepatic recurrence	255 (42.3)	224 (54.6)	<0.01
Extrahepatic recurrence	42 (7.0)	18 (4.4)	0.08
Early recurrence within 2 years	176 (29.2)	170 (41.5)	<0.01
Late recurrence after 2 years	98 (16.3)	68 (16.6)	0.89

Abbreviations: AST, Aspartate aminotransferase; ALT, Alanine aminotransferase; HBV, hepatitis virus type B; HCV, hepatitis virus type C; NBNC, non‐B, non‐C hepatitis; AFP, alpha‐fetoprotein; DCP, des‐γ‐carboxy prothrombin; ICG‐R15, indocyanine green retention rate at 15 minutes.

### 3.2 Comparison between FIB3 index and FIB4 index for prediction of liver fibrosis

**FIGURE 2:**
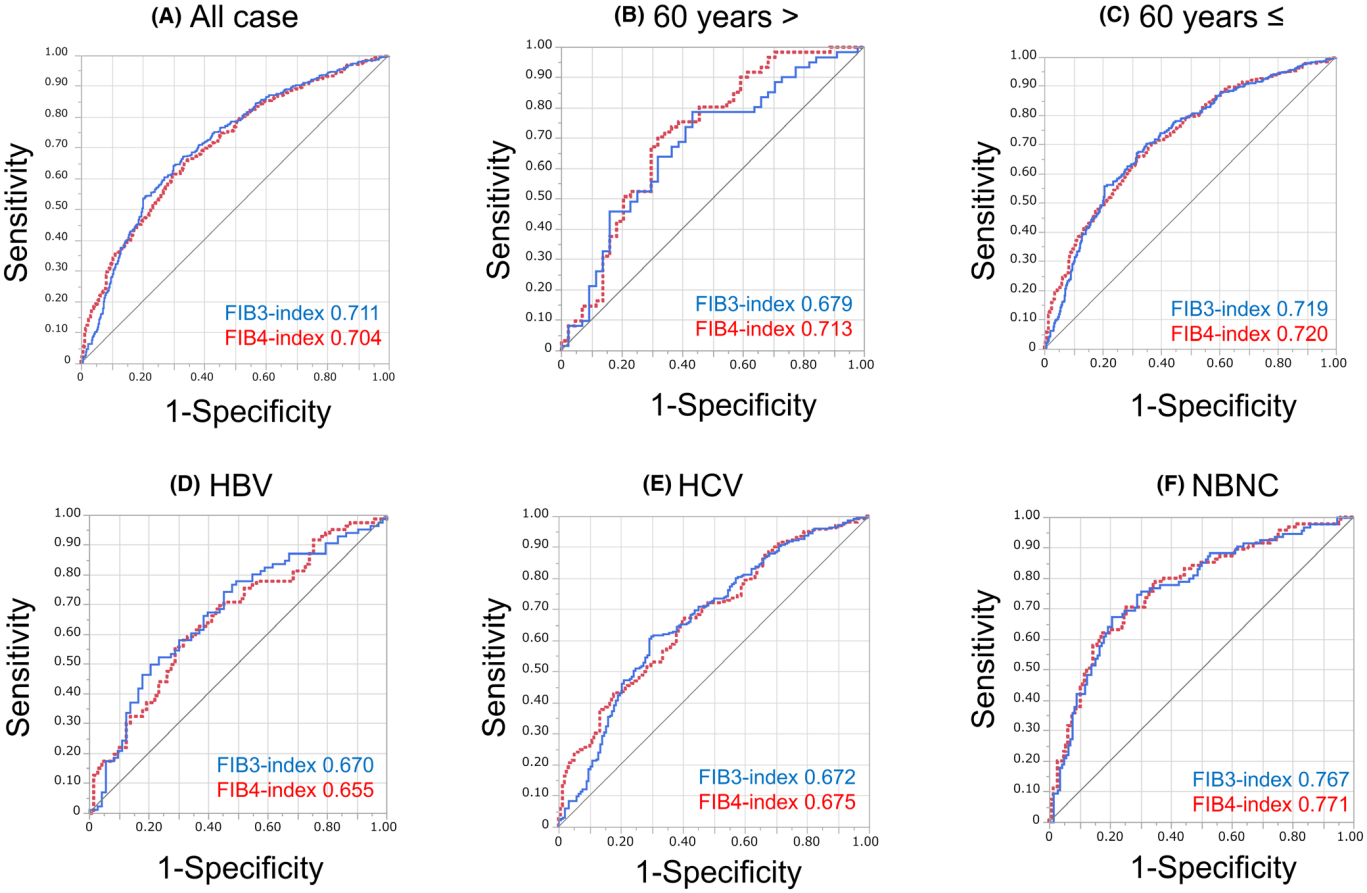
The AUROC of the noninvasive liver fibrosis markers for liver fibrosis. (A) All cases. (B) Patients aged <60 years. (C) Patients aged ≥60 years. (D) Patients with HBV‐related HCC. (E) Patients with HCV‐related HCC. (F) Patients with NBNC‐related HCC.

The stratified analysis revealed the following AUROC values for liver fibrosis (grade III or higher): FIB‐3 index (0.711) and FIB‐4 index (0.704) (Figure 2A, N.S.). Furthermore, stratified analysis according to age revealed the following AUROC values for liver fibrosis (grade III or higher): FIB‐3 index (0.679) and FIB‐4 index (0.713) in younger patients (<60 years) (Figure 2B, N.S.), FIB‐3 index (0.719), and FIB‐4 index (0.720) in elderly patients (≥60 years) (Figure 2C, N.S.). FIB‐3 index (0.679) and FIB‐4 index (0.726) among younger patients (<70 years) (Figure S1A, N.S.), and FIB‐3 index (0.708) and FIB‐4 index (0.720) among elderly patients (≥70 years) (Figure S1B, N.S.). Furthermore, stratified analysis according to etiology revealed the following AUROC values: liver fibrosis (grade III or higher), FIB‐3 index (0.670), and FIB‐4 index (0.655) among patients with HBV‐related HCC (Figure 2D, N.S.), FIB‐3 index (0.672), FIB‐4 index (0.675) among HCV‐related HCC patients (Figure 2E, N.S.), FIB‐3 index (0.767), and FIB‐4 index (0.771) among NBNC‐related HCC patients (Figure 2F, N.S.).

**FIGURE 3:**
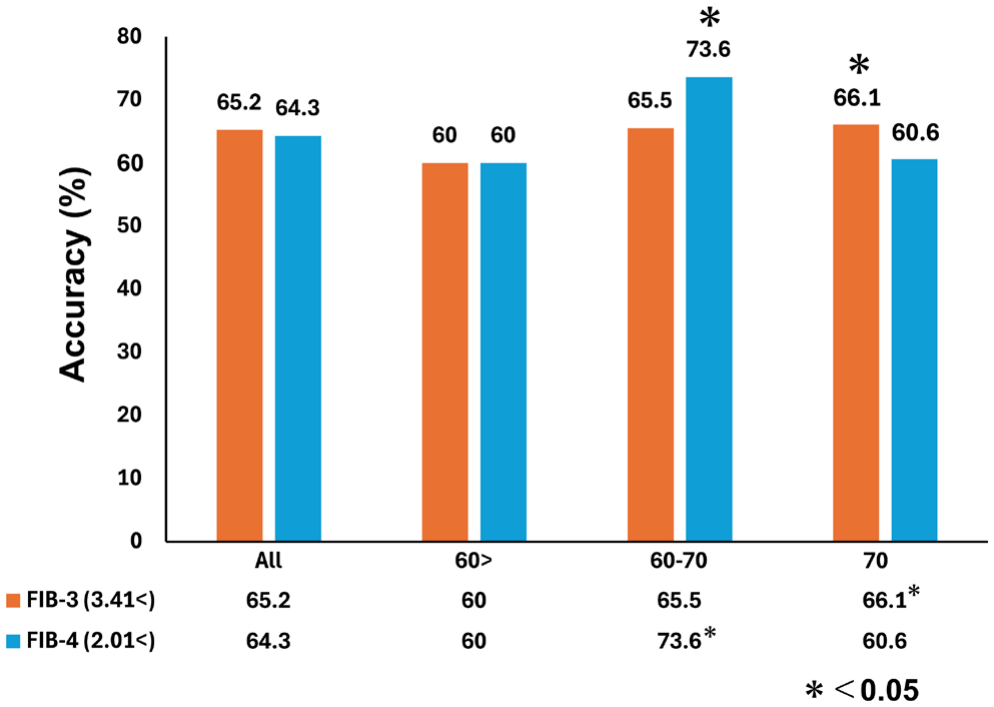
The accuracies of the noninvasive liver fibrosis markers for liver fibrosis. Accuracy is defined as the proportion of correctly classified samples out of the total sample size. To compare the predictive accuracy of liver fibrosis between the two models, McNemar's test was employed. A *p*‐value of <0.05 was considered statistically significant.

Furthermore, the diagnostic accuracies of the FIB‐3 and FIB‐4 indices for liver fibrosis were compared using single cut‐offs^7^ (Figure 3). Overall, the diagnostic performances of the two indices were comparable across all patients (65.2% vs. 64.3%, *p* = 0.52). However, in patients aged 60–69 years, the FIB‐4 index demonstrated significantly higher diagnostic accuracy compared to the FIB‐3 index (65.5% vs. 73.6%, *p* = 0.01). Conversely, among patients aged ≥70 years, the FIB‐3 index exhibited superior diagnostic performance (66.1% vs. 60.6%, *p* = 0.04).

### 3.3 Overall outcomes of all patients

**FIGURE 4:**
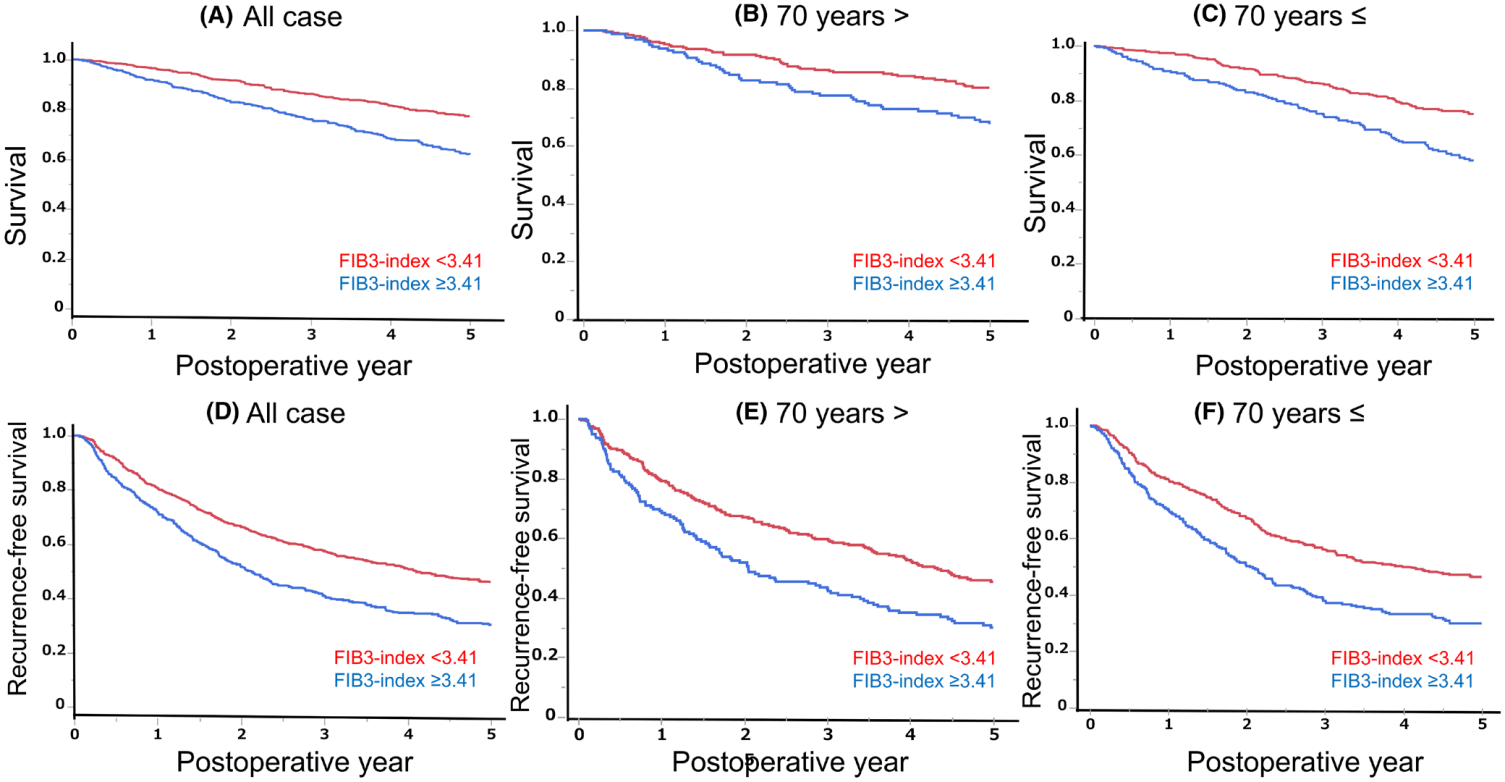
The 5‐year OS and RFS according to the FIB‐3 index before the propensity matching. (A) 5‐year OS in all cases. (B) 5‐year OS in patients aged <70 years. (C) 5‐year OS Patients aged ≥70 years. (D) 5‐year OS in all cases. (E) 5‐year OS in patients aged <70 years. (F) 5‐year OS Patients aged ≥70 years.

The characteristics of the patients, according to the FIB‐3 index, are summarized in Table 1. The FIB‐3 index can be stratified into two distinct grades, each demonstrating unique 5‐year patient survival. The cutoff value was set at 3.41.^7^ Patients in the low fibrosis group (<3.41, *N* = 603) had 1‐, 3‐, and 5‐year survival rates of 96.3%, 86.2%, and 77.2%, respectively, whereas those in the high fibrosis group (≥3.41, *N* = 410) had rates of 91.9%, 76.0%, and 61.7%, respectively (*p* < 0.01, Figure 4A).

Subsequently, in a sub‐analysis based on age, fibrosis was stratified into three distinct grades with cut‐off values,^7^ each demonstrating a unique 5‐year survival. For patients younger than 70 years in the low fibrosis group (<3.41, *N* = 252), the 5‐year survival rate was 80.1%. In contrast, the high fibrosis group (≥3.41, *N* = 160) had a significantly lower rate of 67.5% (*p* < 0.01, Figure 4B). Similarly, for patients aged 70 years and older, the low fibrosis group (<3.41, *N* = 351) had a 5‐year survival rate of 75.0%. However, the high fibrosis group (≥3.41, *N* = 250) exhibited a worse prognosis, with a 5‐year overall survival rate of 58.0% (*p* < 0.01, Figure 4C).

The FIB‐3 index can be classified into two grades, each demonstrating distinct 5‐year recurrence‐free survival (RFS). In the low fibrosis group (<3.41, *N* = 603), the 1‐, 3‐, and 5‐year RFS rates were 80.3%, 57.3%, and 45.9%, respectively. In contrast, the high fibrosis group (≥3.41, *N* = 410) had lower RFS rates of 71.7%, 40.7%, and 30.2% (*p* < 0.01, Figure 4D). For patients younger than 70 years, the 5‐year RFS was 45.4% in the low fibrosis group (<3.41, *N* = 252), while it was significantly lower at 30.0% in the high fibrosis group (≥3.41, *N* = 160) (*p* < 0.01, Figure 4E). Similarly, in patients aged 70 years and older, the 5‐year RFS was 46.3% in the low fibrosis group (<3.41, *N* = 351) and 29.9% in the high fibrosis group (≥3.41, *N* = 250) (*p* < 0.01, Figure 4F).

### 3.4 Risk factors for outcomes using multivariate analysis

In Table 2, the risk factors for 5‐year OS and 5‐year RFS were identified through multivariate analysis. The FIB‐3 index was an independent risk factor for 5‐year OS (HR: 1.10, 95% CI: 1.04–1.17, *p* < 0.01) and 5‐year RFS (HR: 1.07, 95% CI: 1.02–1.12, *p* < 0.01). In addition, across all age groups, the FIB‐3 index demonstrated varying significance for predicting 5‐year RFS as a sub‐analysis (Table 3). In patients aged 60–69 years, FIB‐3 index did not show a significant association with 5‐year RFS (HR: 1.01, 95% CI: 0.93–1.10, *p* = 0.73). Similarly, among patients younger than 60 years, FIB‐3 index was not significantly associated with recurrence risk (HR: 1.07, 95% CI: 0.93–1.25, *p* = 0.33). However, in patients 70 years and older, a significant relationship was observed (HR: 1.09, 95% CI: 1.03–1.15, *p* < 0.01), suggesting that higher FIB‐3 index are associated with increased recurrence risk in this age group. The FIB‐4 index was also detected as the significance for predicting 5‐year OS and RFS (Table S1).

**TABLE 2:** Risk factors for outcomes using the multivariate analysis.

Factors	5‐year OS			5‐year RFS		
	HR	95% CI	*p*‐Value	HR	95% CI	*p*‐Value
Gender, male	1.06	0.77–1.46	0.72	1.31	1.04–1.64	0.02
Age, years	1.02	1.00–1.03	0.04	1.00	0.99–1.01	0.44
Etiology (NBNC: Ref)	(1)			(1)		
HBV	0.89	0.58–1.36	0.58	0.84	0.71–1.26	0.68
HCV	1.02	0.76–1.38	0.88	1.27	1.03–1.56	0.03
Alcohol	1.10	0.69–1.76	0.68	0.91	0.64–1.29	0.60
T‐bil, mg/dL	1.01	0.70–1.45	0.97	1.03	0.81–1.31	0.80
Albumin, g/L	0.49	0.37–0.64	<0.01	0.58	0.48–0.71	<0.01
ICG‐R15, %	1.01	0.99–1.02	0.14	1.00	0.99–1.01	0.45
Log DCP, mAU/mL	1.35	1.19–1.52	<0.01	1.28	1.17–1.40	<0.01
Log AFP, ng/mL	1.16	1.03–1.30	0.01	1.13	1.04–1.23	<0.01
Anatomical resection, yes	1.23	0.91–1.66	0.17	0.97	0.80–1.18	0.79
Tumor size, mm	1.00	1.00–1.00	<0.01	1.00	0.99–1.00	0.24
Multiple tumors, yes	1.37	1.06–1.79	0.02	1.96	1.64–2.24	<0.01
Vascular invasion, yes	1.35	1.04–1.75	0.02	1.28	1.07–1.53	<0.01
FIB‐3 index	1.10	1.04–1.17	<0.01	1.07	1.02–1.12	<0.01

Abbreviations: HBV, hepatitis B Virus; HCV, hepatitis C Virus; ICG‐R15, indocyanine green retention rate at 15 minutes; NBNC, non‐B, non‐C hepatitis.

**TABLE 3:** Risk factors for 5Y‐RFS due to patients age using the multivariate analysis.

Factors	60 y<			60–69y			≥70y		
	HR	95% CI	*p*‐Value	HR	95% CI	*p*‐Value	HR	95% CI	*p*‐Value
Gender, male	1.12	0.50–2.53	0.78	1.27	0.82–1.98	0.29	1.34	0.99–1.79	0.05
Age, years	1.02	0.97–1.08	0.50	1.08	1.01–1.14	0.02	1.02	0.99–1.04	0.18
Etiology (NBNC: Ref)	(1)			(1)			(1)		
HBV	0.45	0.18–1.15	0.09	1.00	0.61–1.64	0.96	1.00	0.64–1.57	0.99
HCV	0.78	0.28–2.16	0.63	1.14	0.76–1.73	0.56	1.34	1.03–1.74	0.03
Alcohol	0.16	0.02–1.63	0.13	0.71	0.38–1.33	0.25	1.13	0.73–1.76	0.59
T‐bil, mg/dL	0.87	0.45–1.63	0.67	0.92	0.56–1.45	0.74	1.10	0.79–1.54	0.57
Albumin, g/L	0.65	0.37–1.15	0.14	0.51	0.36–0.72	<0.01	0.59	0.45–0.77	<0.01
ICG‐R15, %	1.04	1.01–1.07	0.01	1.01	0.99–1.02	0.48	1.00	0.99–1.01	0.84
Log DCP, mAU/mL	1.54	1.03–2.36	0.04	1.37	1.14–1.64	<0.01	1.22	1.08–1.37	<0.01
Log AFP, ng/mL	0.78	0.57–1.07	0.13	1.28	1.18–1.51	<0.01	1.15	1.03–1.28	0.02
Anatomical resection, yes	1.38	0.77–2.49	0.28	0.97	0.65–1.46	0.90	0.90	0.70–1.17	0.43
Tumor size, mm	1.01	0.99–1.03	0.27	1.00	0.99–1.00	0.32	1.00	0.99–1.00	0.47
Multiple tumors, yes	2.31	1.25–4.31	<0.01	1.60	1.13–2.27	<0.01	2.04	1.62–2.58	<0.01
Vascular invasion, yes	1.42	0.77–2.63	0.26	1.16	0.82–1.63	0.40	1.33	1.05–1.69	0.02
FIB‐3 index	1.07	0.93–1.25	0.33	1.01	0.93–1.10	0.73	1.09	1.03–1.15	<0.01

Abbreviations: HBV, hepatitis B virus; HCV, hepatitis C virus; ICG‐R15, indocyanine green retention rate at 15 minutes; NBNC, non‐B, non‐C hepatitis.

After the propensity score matching using the detected risk factors for 5‐year RFS, the high FIB‐3 index group had a significantly worse prognosis in all cases (*p* < 0.01, Figure S2A) and the elderly patients over 70 years (*p* < 0.01, Figure S2B). Similar outcomes between the two groups could be seen in other populations (*p* = 0.49. Figure S2C, *p* = 0.15, Figure S2D).

### 3.5 Prognostic value of the FIB‐3 index

Table 4 in the provided text presents the AUROC values for the FIB‐3 and FIB‐4 indices across various patient groups for assessing fibrosis stage (≥F3), 5‐year OS, and 5‐year RFS. The FIB‐4 index generally demonstrated a performance similar to that of the FIB‐3 index, with an overall AUROC of 0.711 compared to 0.704. The FIB‐4 index achieved the highest performance in the NBNC group at 0.771, whereas the lowest was observed in the HBV group at 0.655. Conversely, the FIB‐3 index showed the best performance in the NBNC group at 0.767, but it was the lowest in the HBV group at 0.670.

**TABLE 4:** Predictive value of FIB‐3 index and FIB‐4 index.

	Index	All	AUROC						
			60 y<	60–69y	≥70y	HBV	HCV	NBNC	Alc
Fibrosis (≧F3)	FIB‐3 index	0.711	0.679	0.743	0.708	0.670	0.672	0.767	0.718
	FIB‐4 index	0.704	0.713	0.747	0.720	0.655	0.675	0.771	0.713
5‐year OS	FIB‐3 index	0.600	0.674	0.581	0.599	0.570	0.641	0.547	0.568
	FIB‐4 index	0.584	0.639	0.556	0.582	0.530	0.625	0.532	0.565
5‐year RFS	FIB‐3 index	0.593	0.616	0.560	0.607	0.499	0.604	0.589	0.576
	FIB‐4 index	0.583	0.577	0.557	0.604	0.532	0.608	0.569	0.582

Abbreviations: AUROC, area under the receiver operating characteristic; HBV, hepatitis B virus; HCV, hepatitis C virus; NBNC, non‐B, non‐C hepatitis; OS, overall survival; RFS, recurrence‐free survival.

Regarding 5‐year OS, both indices showed comparatively lower AUROC values than fibrosis prediction. The FIB‐3 index had an overall AUROC of 0.600, with the best performance in the younger group at 0.674 and the worst in the NBNC group at 0.547. Regarding 5‐year RFS, the FIB‐3 index had an overall AUROC of 0.593, with the best performance in the younger group at 0.616 and the worst in the HBV group at 0.499.

**FIGURE 5:**
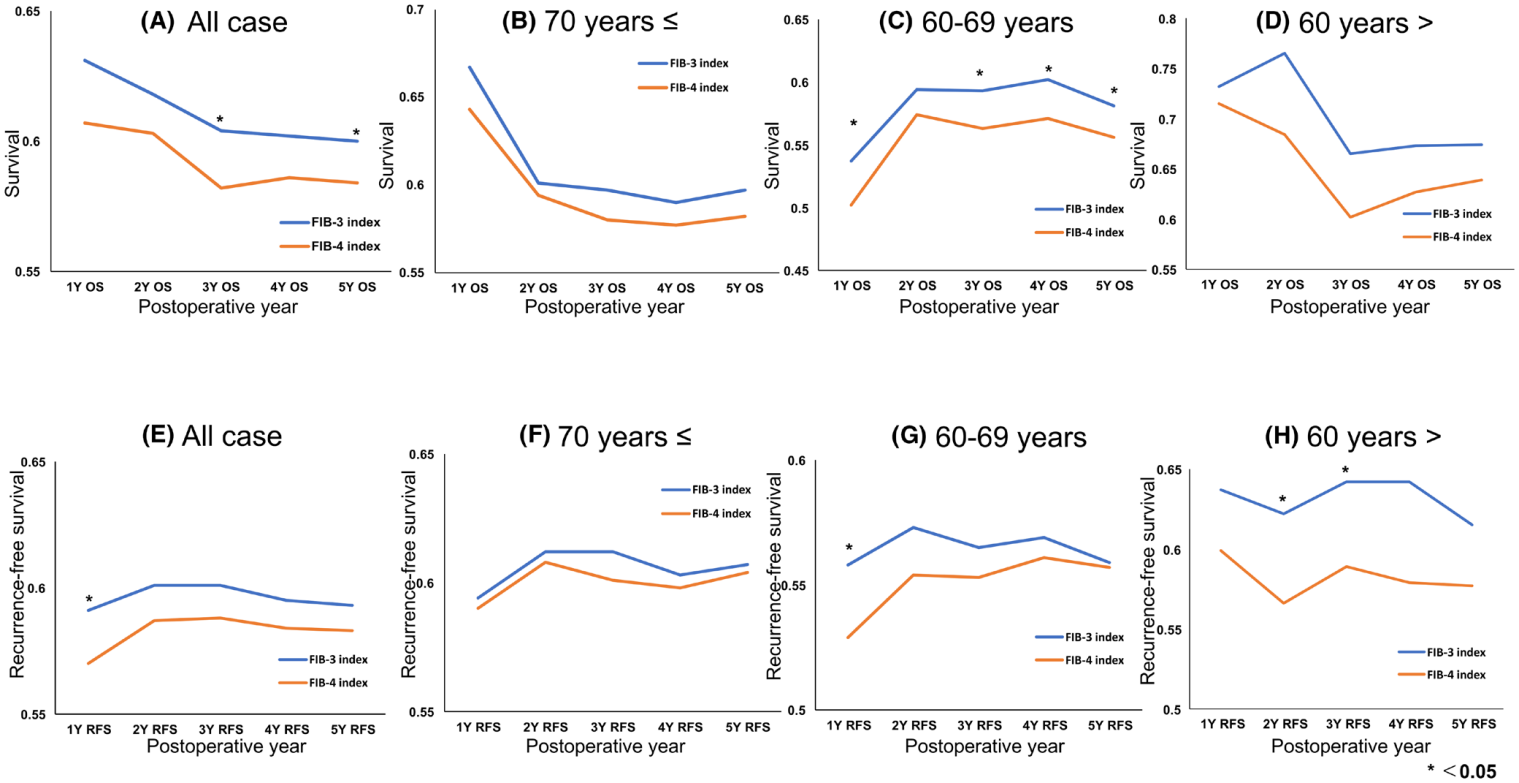
The time‐dependent AUROC analysis for predicting the OS and RFS. (A) 5‐year OS in all cases. (B) 5‐year OS in patients aged ≥70 years. (C) 5‐year OS in patients aged 60–69 years. (D) 5‐year OS Patients aged <60 years. (E) 5‐year RFS in all cases. (F) 5‐year RFS in patients aged <60 years. (G) 5‐year RFS in patients aged 60–69 years. (H) 5‐year RFS Patients aged ≥70 years. The AUROC was plotted for each year of the 1‐ to 5‐year prognosis. The AUROC comparisons between models for predicting liver fibrosis and postoperative outcomes were performed using the DeLong test. A *p*‐value of <0.05 was considered statistically significant.

The prognostic value of the FIB‐3 and FIB‐4 indices for OS and RFS was assessed using time‐dependent AUROC analysis (Figure 5A–H). The analysis revealed a complex trajectory in prognostic accuracy. Across all age groups, the FIB‐3 index demonstrated superior prognostic performance compared to the FIB‐4 index for both OS and RFS (Figure 5A,E), with significantly higher predictive ability for 3‐year OS, 5‐year OS, and 1‐year RFS.

In patients aged over 70, the predictive ability of the FIB‐3 index for 5‐year OS showed a trend toward superiority (*p* = 0.06), whereas the predictive performance for RFS was nearly identical between the two indices (Figure 5B,F). In the 60–69‐year‐old group, the FIB‐3 index exhibited significantly greater OS predictive ability than the FIB‐4 index (Figure 5C). Additionally, in patients aged 60 and younger, the FIB‐3 index demonstrated higher predictive ability for RFS (Figure 5H).

## 4 DISCUSSION

This multicenter study demonstrated that the novel non‐invasive fibrosis assessment index, the FIB‐3 index, is effective in predicting advanced liver fibrosis (F3 or higher) and prognosis in patients undergoing liver resection for HCC. The predictive ability of the FIB‐3 index, which utilizes liver fibrosis as a continuous variable, was comparable to that of the FIB‐4 index across all age groups and hepatic backgrounds. However, when using the effective cut‐off levels, the predictive accuracy of liver fibrosis FIB‐3 index can be superior among the patients over 70 years. For OS, the FIB‐3 index demonstrated superior prognostic performance compared to the FIB‐4 index, particularly in the overall cohort and among patients aged 60–69 years. While the predictive utility of the FIB‐3 index for RFS was limited in elderly patients, it remained clinically relevant in the overall population, similar to the FIB‐4 index. Nobly, the application of appropriate cutoff values allowed the FIB‐3 index to facilitate effective risk stratification for 5‐year RFS in elderly patients (≥70 years). These findings highlight the significance of incorporating the FIB‐3 index, which excludes age‐related factors, in recurrence risk models.

The increasing prevalence of chronic liver disease has necessitated the development of clinical tools for risk stratification, personalized treatment, and monitoring of HCC. Consequently, noninvasive markers and models for liver fibrosis have been developed.^14^ Scoring models that employ serum markers commonly assessed for hepatic impairment, such as the FIB‐4 index and AST/platelet number ratio index (APRI), have been developed for patients with chronic hepatitis C viral infection.^13^ These models have since been applied to patients with other hepatic disorders including NBNC.^6^, ^7^, ^15^, ^16^ The FIB‐4 index was not initially designed to diagnose liver fibrosis in elderly patients,^7^ but surveillance is needed for elderly patients in the real world. The diagnostic accuracy of the FIB‐4 index for liver fibrosis, employing a single cutoff value, decreased for individuals aged ≥70 years, as shown in Figure 3. While the accuracy of the FIB‐4 index could be improved by employing different cutoff values for various age groups,^17^ this approach would be infeasible in clinical settings because of its complexity and the difficulty in determining optimal cutoff values for different age groups. Our study demonstrated that the prognostic risk of liver fibrosis and HCC following surgery in elderly patients can be effectively stratified using an appropriate cutoff for the FIB‐3 index, independent of age‐related factors. With the increasing number of elderly patients undergoing surgical intervention, the clinical relevance of the FIB‐3 index is expected to grow, further emphasizing its utility in risk assessment, postoperative management, and recurrence screening.

In this study, the high FIB‐3 group exhibited a more aggressive biological phenotype. Notably, despite having significantly smaller tumor diameters, this group demonstrated a higher proportion of multiple tumors and elevated AFP levels, leading to a significantly increased risk of intrahepatic and early recurrence. The aggressive biological phenotype observed in patients with high fibrosis has been consistently reported across different study populations, further seeking its clinical significance.^3^, ^5^, ^18^ The primary reason for this phenomenon is that patients with liver cirrhosis or advanced liver fibrosis are considered a high‐risk group for HCC and are recommended to undergo regular imaging assessments along with tumor marker measurements. These surveillance strategies facilitate the early detection of HCC at smaller tumor sizes, leading to a higher proportion of reported cases with smaller tumor diameters. Additionally, the functional contribution of liver fibrosis to HCC development and aggressiveness is well established, with multiple mechanisms implicated in its initiation and progression. In a fibrotic liver, the upregulation of cytokines promotes epithelial‐mesenchymal transition, enhancing the invasive and metastatic potential of HCC cells.^19^, ^20^, ^21^, ^22^ Furthermore, inflammatory cytokines and chemokines contribute to immunosuppression by inhibiting immune cell activity, thereby attenuating the liver's anti‐tumor immune response.^23^, ^24^, ^25^ These comprehensive mechanisms include extracellular matrix deposition by hepatic stellate cells, the release of growth factors and cytokines, fibrosis‐induced alterations in signaling pathways, and immunosuppressive effects, all of which collectively contribute to the acceleration of tumorigenesis and tumor biology.^26^

Among patients with HCC, those with underlying advanced liver fibrosis have been reported to have a 6%–15% higher annual risk of de novo recurrence compared to those who underwent HCC resection from a normal liver.^3^ In addition, conventional treatments such as radiofrequency ablation, chemotherapy, and liver resection often fall short because they do not eradicate the cancerous environment within the liver.^27^ The after‐DAA recommendation for surveillance (ADRES) score,^28^, ^29^ based on sex, FIB‐4 index, and α‐fetoprotein, were used to predict the development of HCC in HCV patients. A modified ADRES score has been developed and effectively evaluated for its ability to predict HCC development in comparison to the original ADRES score, with the FIB‐4 index replaced by the FIB‐3 index.^30^ In older patients, the conventional FIB‐4 index may not accurately assess the progression of liver fibrosis.^30^ The FIB‐3 index, which excludes the age factor, should be replaced in the field of surgery because 59.3% were aged ≥70 years in this cohort for liver resection. We hypothesized that the FIB‐3 index, which predicts liver fibrosis, could also be used to predict the prognosis after HCC hepatectomy. Previous reports have shown that patients with a higher FIB‐4 index demonstrated inferior OS and RFS after hepatic resection for HCC.^4^, ^5^, ^31^, ^32^, ^33^, ^34^ Our result also indicated that a higher FIB‐3 index was related to worse OS and RFS even after the propensity matching, as depicted in Figures 4 and S2. The superiority of the FIB‐3 index over the FIB‐4 index in predicting OS and RFS can be attributed to the limited prognostic impact of age on 5‐year RFS. Specifically, in patients aged ≥70 years and those <60 years, age was not identified as an independent risk factor. Furthermore, the HR for the FIB‐3 index, which excludes age as a variable (HR 1.07), was higher than that of the FIB‐4 index (HR 1.04), suggesting greater predictive strength. Given that individuals aged ≥70 years constituted 60% of the study population, the overall predictive advantage of the FIB‐3 index was likely driven by its applicability across the entire cohort. Although the FIB‐3 index demonstrated superior performance for 5‐year RFS compared to the FIB‐4 index across all cases, its AUROC was still modest at approximately 0.60, suggesting that its predictive utility could be enhanced by integrating it with tumor markers and pathological findings.^35^ Furthermore, a higher FIB‐3 index was identified as an independent risk factor for 5‐year RFS in elderly patients, but not in younger individuals. The predictive value of the FIB‐3 index was shown to be more clinically significant in older patients. These findings underscore the clinical importance of incorporating the FIB‐3 index, independent of age factors, in recurrence risk assessment models.

This study has several limitations that warrant consideration. First, the multicenter database used in this study did not include patients treated without surgery. As a result, we were unable to assess the prognostic significance of the FIB‐3 and FIB‐4 indices in patients with nonsurgical HCC or the diagnostic value of liver fibrosis in patients who received nonsurgical treatment. The analysis of post‐relapse treatment details was limited due to a retrospective multicenter study. It is important to note that this study's retrospective design made it challenging to identify MASLD in NBNC patients based on a strict definition, necessitating careful interpretation of the findings.

## 5 CONCLUSION

The FIB‐3 index served as an effective alternative to the FIB‐4 index in assessing liver fibrosis among aged patients, and it effectively stratified the likelihood of the 5‐year outcomes when utilized in conjunction with a specific cut‐off after initial hepatectomy for HCC.

## AUTHOR CONTRIBUTIONS

**Yuki Imaoka:** Conceptualization; formal analysis; funding acquisition; investigation; methodology; writing – original draft. **Masahiro Ohira:** Conceptualization; funding acquisition; supervision; writing – original draft; writing – review and editing. **Tsuyoshi Kobayashi:** Writing – review and editing. **Naruhiko Honmyo:** Data curation. **Michinori Hamaoka:** Data curation. **Takashi Onoe:** Data curation. **Daisuke Takei:** Data curation. **Koichi Oishi:** Data curation. **Tomoyuki Abe:** Data curation; writing – review and editing. **Toshihiro Nakayama:** Formal analysis. **Miho Akabane:** Formal analysis. **Kazunari Sasaki:** Formal analysis. **Hideki Ohdan:** Funding acquisition; supervision; writing – review and editing.

## FUNDING INFORMATION

This work was partly supported by JSPS KAKENHI (grant numbers 22 K16535, 23H02981) and AMED (grant number 24fk0210108).

## CONFLICT OF INTEREST STATEMENT

Hideki Ohdan is an editorial board member of *Annals of Gastroenterological Surgery*.

## ETHICS STATEMENT

Approval of the research protocol: The study protocol was approved by the ethics committee of our hospital (E‐2057).

Informed Consent: This study conformed to the provisions of the Declaration of Helsinki. The need for written informed consent was waived because of the retrospective nature of the study. The opt‐out method for obtaining patient consent was used by all institutions.

Registry and the Registration No. of the study/trial: N/A.

Animal Studies: N/A.

Research involving recombinant DNA: N/A.

## Supporting information


Figure S1.

Figure S2.

Table S1.

## Data Availability

The datasets used and/or analyzed during the current study are available from the corresponding author on reasonable request.
