# Supplementary figures and images for: FIB‐3 index as a novel age‐independent predictor of liver fibrosis and prognosis in hepatocellular carcinoma patients undergoing hepatectomy
Source: Ann Gastroenterol Surg. 2025 Apr 13;9(5):1055–65. doi: 10.1002/ags3.70010 (PMC12414608; doi:10.1002/ags3.70010)

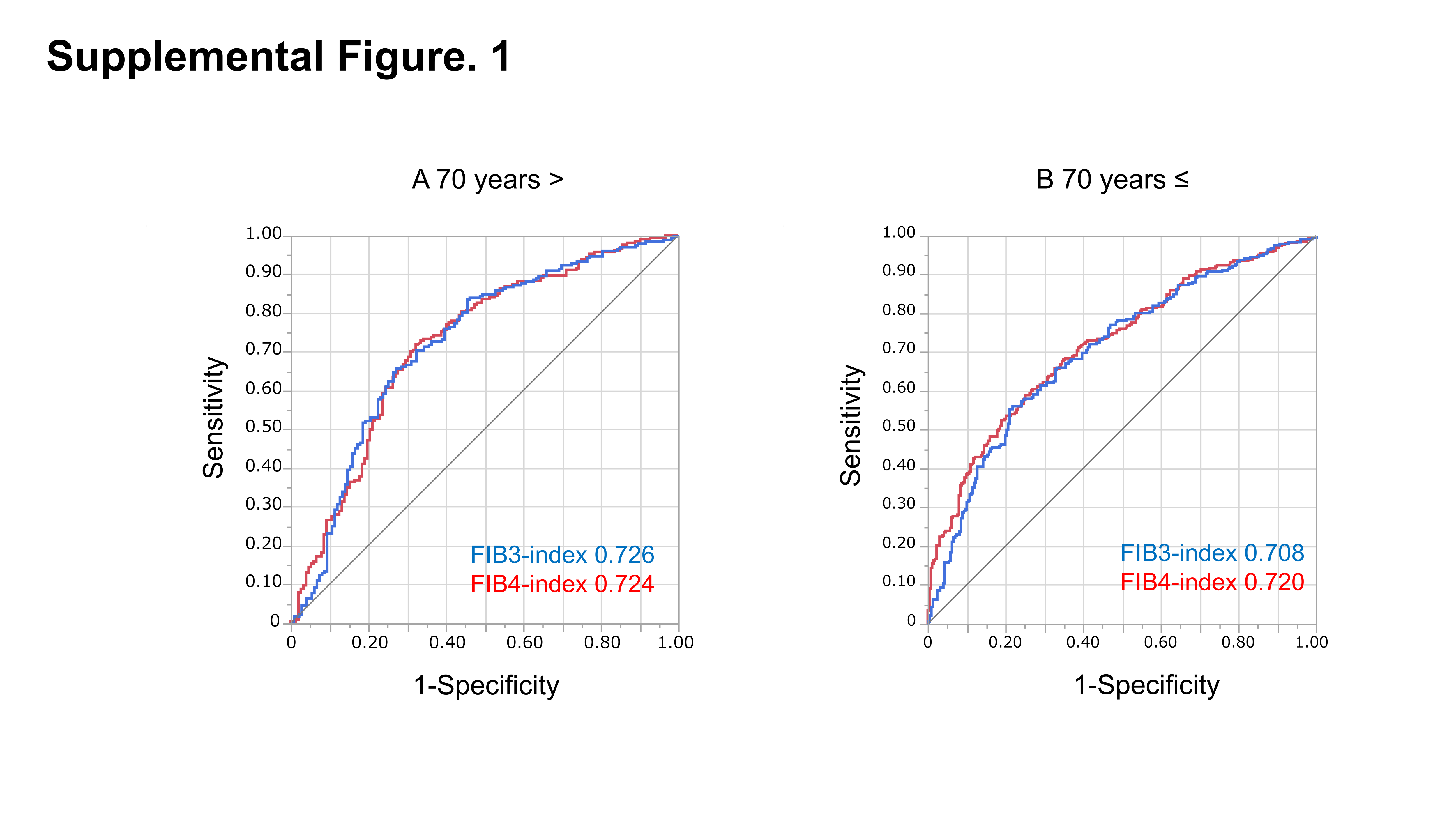

Supplement: Supplementary file 1 — Figure S1. [file AGS3-9-1055-s003.tif]

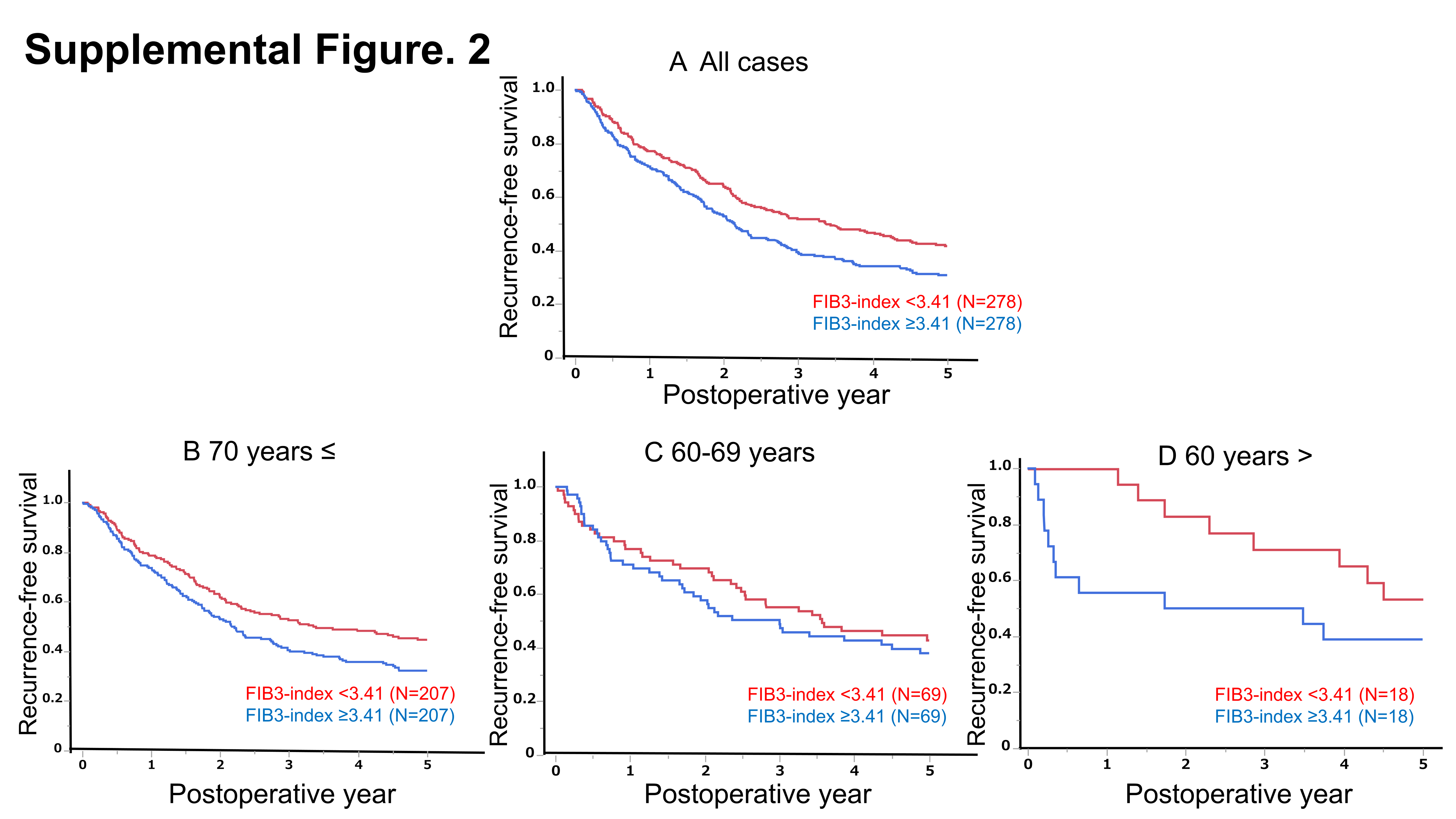

Supplement: Supplementary file 2 — Figure S2. [file AGS3-9-1055-s001.tif]
