# Supplementary material for: FIB‐3 index as a novel age‐independent predictor of liver fibrosis and prognosis in hepatocellular carcinoma patients undergoing hepatectomy
Source: Ann Gastroenterol Surg. 2025 Apr 13;9(5):1055–65. doi: 10.1002/ags3.70010 (PMC12414608; doi:10.1002/ags3.70010)
Supplement: Supplementary file 3 — Table S1. [file AGS3-9-1055-s002.docx]

| Supplemental Table 1. Risk factors for outcomes using the multivariate analysis | | | | | | |
| --- | --- | --- | --- | --- | --- | --- |
| Factors | 5-year OS | | | 5-year RFS | | |
|  | HR | 95% CI | p-value | HR | 95% CI | p-value |
| Gender, male | 1.05 | 0.76-1.45 | 0.76 | 1.29 | 1.02-1.61 | **0.03** |
| Age, years | - | - | **-** | - | - | - |
| Etiology  (NBNC: Ref)  HBV  HCV  Alcohol | (1)  0.78  1.02  1.10 | 0.52-1.17  0.76-1.37  0.69-1.76 | 0.23  0.88  0.68 | (1)  0.92  1.28  0.91 | 0.70-1.20  1.04-1.57  0.65-1.29 | 0.53  **0.02**  0.61 |
| T-bil, mg/dl | 1.01 | 0.65-1.33 | 0.73 | 1.03 | 0.81-1.31 | 0.77 |
| Albumin, g/L | 0.51 | 0.39-0.67 | **<0.01** | 0.58 | 0.48-0.71 | **<0.01** |
| ICG-R15, % | 1.01 | 0.99-1.02 | 0.13 | 1.00 | 0.99-1.01 | 0.32 |
| Log DCP, mAU/ml | 1.37 | 1.20-1.54 | **<0.01** | 1.29 | 1.18-1.41 | **<0.01** |
| Log AFP, ng/ml | 1.15 | 1.03-1.29 | **0.02** | 1.14 | 1.05-1.24 | **<0.01** |
| Anatomical resection, yes | 1.24 | 0.92-1.68 | 0.14 | 0.98 | 0.81-1.19 | 0.82 |
| Tumor size, mm | 1.00 | 1.00-1.00 | **0.02** | 1.00 | 0.99-1.00 | 0.24 |
| Multiple tumors, yes | 1.37 | 1.05-1.77 | **0.02** | 1.96 | 1.64-2.24 | **<0.01** |
| Vascular invasion, yes | 1.38 | 1.07-1.79 | **0.01** | 1.30 | 1.08-1.55 | **<0.01** |
| FIB-4 index | 1.08 | 1.04-1.13 | **<0.01** | 1.04 | 1.01-1.07 | **0.01** |

indocyanine green retention rate at 15 minutes; ICG-R15, HBV: Hepatitis B Virus, HCV: Hepatitis C Virus, NBNC: Non-B, Non-C Hepatitis
